# Monocyte subsets predict mortality after cardiac arrest

**DOI:** 10.1002/JLB.5A0420-231RR

**Published:** 2020-10-05

**Authors:** Konstantin A. Krychtiuk, Max Lenz, Bernhard Richter, Philipp. J. Hohensinner, Stefan P. Kastl, Andreas Mangold, Kurt Huber, Christian Hengstenberg, Johann Wojta, Gottfried Heinz, Walter S. Speidl

**Affiliations:** ^1^ Division of Cardiology Department of Internal Medicine II Medical University of Vienna Vienna Austria; ^2^ Ludwig Boltzmann Cluster for Cardiovascular Research Vienna Austria; ^3^ 3rd Medical Department Wilhelminen Hospital Vienna Austria; ^4^ Core Facilities Medical University of Vienna Vienna Austria

**Keywords:** cardiac arrest, innate immunity, monocyte subsets, monocytes

## Abstract

After successful cardiopulmonary resuscitation (CPR), many patients show signs of an overactive immune activation. Monocytes are a heterogeneous cell population that can be distinguished into 3 subsets by flow cytometry (classical monocytes [CM: CD14^++^CD16^‐^], intermediate monocytes [IM: CD14^++^CD16^+^CCR2^+^] and non‐classical monocytes [NCM: CD14^+^CD16^++^CCR2^‐^]). Fifty‐three patients admitted to the medical intensive care unit (ICU) after cardiac arrest were included. Blood was taken on admission and after 72 h. The primary endpoint of this study was survival at 6 months and the secondary endpoint was neurological outcome as determined by cerebral performance category (CPC)‐score at 6 months. Median age was 64.5 (49.8‐74.3) years and 75.5% were male. Six‐month mortality was 50.9% and survival with good neurological outcome was 37.7%. Monocyte subset distribution upon admission to the ICU did not differ according to survival. Seventy‐two hours after admission, patients who died within 6 months showed a higher percentage of the pro‐inflammatory subset of IM (8.3% [3.8‐14.6]% vs. 4.1% [1.5–8.2]%; *P* = 0.025), and a lower percentage of CM (87.5% [79.9–89.0]% vs. 90.8% [85.9–92.7]%; *P* = 0.036) as compared to survivors. In addition, IM were predictive of outcome independent of time to ROSC and witnessed cardiac arrest, and correlated with CPC‐score at 6 months (*R* = 0.32; *P* = 0.043). These findings suggest a possible role of the innate immune system in the pathophysiology of post cardiac arrest syndrome.

## Graphical Abstract

In patients admitted to an ICU after surviving a cardiac arrest, an increase of intermediate monocytes after 72 hours was associated with poor outcome.

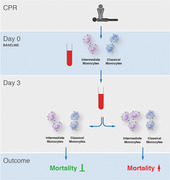

**Abbreviations**
AMI: acute myocardial infarction
APACHE II: Acute physiology and chronic health evaulation
APC‐H7: allophycocyanin
BMI: body mass index
CA: cardiac arrest
CCR‐2: C‐C chemokine receptor‐2
CD: cluster of differentiation
CI: confidence interval
CM: classical monocytes
CPC: cerebral performance category
CPR: cardiopulmonary resuscitation
CRP: C‐reactive protein
ERC: European Resucitation Council
FACS: fluorescence activated cell sorting
HR: hazard ratio
ICU: intensive care unit
IM: intermediate monocytes
IQR: interquartile range
NCM: non‐classical monocytes
OHCA: out of hospital cardiac arrest
PCAS: post cardiac arrest sysndrome
PCT: procalcitonin
PE: phycoerythrin
PerCP: peridinin chlorphyll protein
ROS: reactive oxygen species
ROSC: return of spontaneous circulation
RPM: revolutions per minute
TTM: therapeutic temperature management

## 1 INTRODUCTION

Out of hospital cardiac arrest (OHCA) is one of the leading causes of death in the Western world affecting ∼86–98 per 100,000 inhabitants per year with only 6–10% of patients surviving to hospital discharge.^1^, ^2^

The underlying pathophysiological processes occurring after CA have been termed post cardiac arrest syndrome (PCAS).^3^ The main PCAS characteristics include brain injury, myocardial dysfunction, a persistent precipitating pathology that initially caused the CA and a severe systemic ischemia reperfusion syndrome.^4^ The latter is a paradoxical phenomenon describing the injury caused by restoration of blood flow after an ischemic period. This phenomenon was first described in acute myocardial infarction. It is thought to be present and harmful in CA as well in terms of a whole‐body ischemia reperfusion.^5^ After a period of ischemia with lack of oxygen, restoration of blood flow itself may result in oxidative damage caused by the generation of reactive oxygen species (ROS).^6^ Oxidative stress is a strong innate immune system inducer. This systemic inflammatory response may contribute to patient deterioration and ultimately irreversible multi‐organ damage and death. Still, a major cause of the high mortality in patients after CA is the irreversible brain injury.^3^

Monocytes are circulating leukocytes that migrate into tissues and differentiate into Mϕs. As members of the innate immune system, they have an important physiologic role in the first line of defense against pathogens. However, they have also been causally involved in the pathophysiology of chronic and acute inflammatory conditions. As already described more than 3 decades ago, monocytes are a heterogeneous cell population that can be distinguished into at least 3 subsets according to their CD14 and CD16 surface expression pattern.^7^, ^8^ Functions of classical monocytes (CM) include functions such as phagocytosis, while intermediate monocytes (IM) exhibit distinct features including a strong inflammatory reaction upon stimulation.^9^ The subset of non‐classical monocytes (NCM) on the other hand was described as exhibiting a specific “crawling” behavior at the endothelium during physiologic conditions.^10^

Several larger studies have described monocyte subsets as prognostic markers in stable diseases such as chronic kidney disease^11^ and coronary artery disease.^12^ However, there is only limited data regarding the behavior of monocyte subsets in critically ill patients, while data on monocyte subsets after CA is completely lacking.^13^, ^14^, ^15^

Therefore, the aim of the current study was to evaluate the dynamics of circulating monocyte subsets in the pathophysiology and prognosis in survivors of CA admitted to a medical intensive care unit (ICU).

## 2 MATERIALS AND METHODS

### 2.1 Subjects and study design

In this single‐center, prospective observational study, all patients who were admitted to the cardiac ICU of the Department of Internal Medicine II at the Medical University of Vienna (General Hospital of Vienna), between August 2012 and August 2013 after CA were included consecutively. We included all patients admitted to our medical ICU, both out‐of‐hospital CA admitted via the emergency department as well as in‐hospital CA who were admitted from the in‐hospital resuscitation team as described previously.^16^ Patients aged below 18 years and patients after traumatic CA were not included in this study.

The study was approved by the ethical committee of the Medical University of Vienna (EK 1101/2012) and complies with the Declaration of Helsinki. For unconscious patients, the need for informed consent was waived by the ethical committee. All conscious patients gave informed consent.

All patients were treated according to the latest post‐resuscitation guidelines provided by the European Resuscitation Council (ERC) including further diagnostics and therapeutic interventions.^17^ Patients were treated using targeted temperature management for 24 h except for those immediately regaining consciousness following commands and purposeful movements or were suffering from severe hemodynamic instability. For the quantification of disease severity, the acute physiology and chronic health evaluation II (APACHE II)^18^ score was used.

Recorded pre‐ and in‐hospital parameters and patient characteristics including Utstein variables are listed in Table 1. The Glasgow‐Pittsburgh cerebral performance categories (CPC) were used for neurologic status categorization at 6 months after CA. CPC 1–2 was defined as favorable neurological outcome while CPC 3–5 was considered poor neurological outcome.^19^ The main study endpoint was all‐cause mortality at 6 months.

**TABLE 1:** Baseline characteristics

Parameter	All (*n* = 53)	Survivors (*n* = 26)	Non‐survivors (*n* = 27)	*P*‐value
Age (years)	64.5 (49.8‐74.3)	61.4 (45.5‐72.2)	71.5 (60.7‐81.3)	0.047
Male (*n* (%))	40 (75.5%)	23 (79%)	17 (71%)	0.48
BMI (kg/m^2^)	24.7 (23.3‐29.2)	24.6 (22.8‐29.2)	26.6 (23.9‐29.3)	0.4
In‐hospital CA (n (%))	22 (41.5%)	7 (24.1%)	15 (62.5%)	0.007
Witnessed CA (*n* (%))	42 (79.2%)	27 (93%)	15 (62.5%)	0.006
Bystander – CPR (*n* (%))	47 (88.7%)	27 (93.%)	20 (83%)	0.26
Time to ROSC (min)	20 (10‐42.5)	13.5 (7.3‐30.8)	34 (17‐55)	0.001
Shockable rhythm (*n* (%))	28 (52.8%)	19 (65.5%)	9 (37.5%)	0.042
Lactate (mmol/L)	1.9 (1.3‐3.8)	1.6 (1.2‐3.1)	2.1 (1.3‐6.4)	0.056
TTM (n (%))	40 (75.5%)	22 (75%)	18 (75%)	0.94
Serum creatinine (mg/dl)	1 (0.8‐1.5)	0.9 (0.7‐1.1)	1.4 (0.9‐2)	0.002
APACHE II score	25 (20.3‐28)	22 (15.5‐25)	28 (25‐33)	<0.001

BMI, body mass index; CA, cardiac arrest; ROSC, return of spontaneous circulation; TTM, targeted temperature management; APACHE, acute physiology and chronic health evaluation; Data are given as *n* (%) or median (IQR).

### 2.2 Blood sampling

Blood for flow cytometry was drawn within 24 h after admission and 72 h after the first blood sampling from either the arterial or central venous line. After the initial 3 mL of blood had been discarded, blood was drawn into an EDTA‐tube for immediate flow cytometry. Standard laboratory parameters were measured at the central laboratory of the General Hospital of Vienna.

### 2.3 Flow cytometry

**FIGURE 1:**
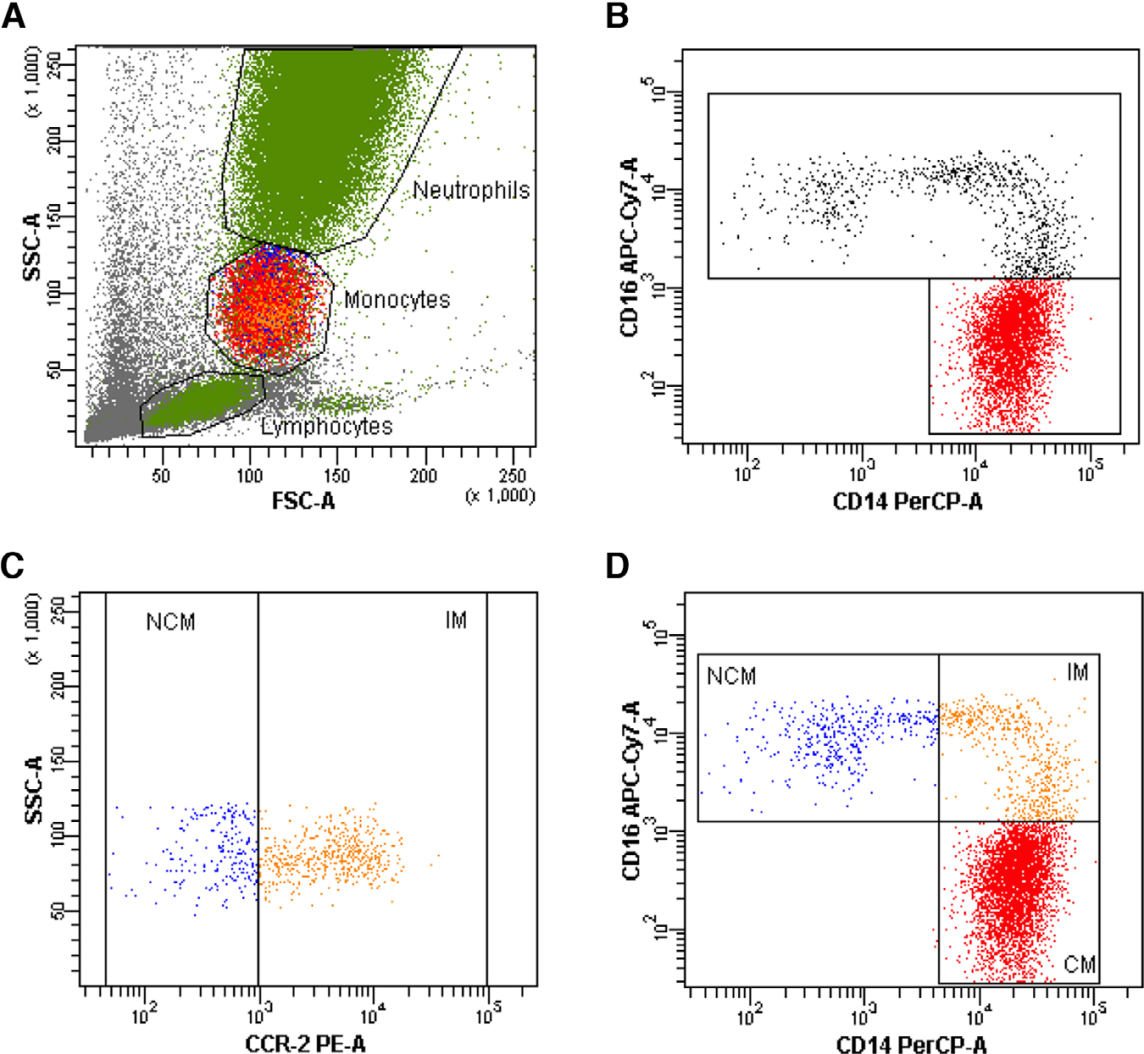
**Gating strategy used for the identification of monocyte subsets**. Monocytes were identified as CD45‐positive cells exhibiting a typical forward and sideward scatter profile (**A**). Monocyte subsets were identified according to their expression profile of CD14 and CD16 (**B**), CD16‐positive monocytes were further distinguished according to their expression of CCR‐2 (**C**). Monocyte subsets were identified as classical monocytes (CM: CD14^++^CD16^+^); intermediate monocytes (IM: CD14^++^CD16^+^CCR2^+^) and non‐classical monocytes (NCM: CD14^+^CD16^++^CCR2^‐^) (**D**)

Whole blood flow cytometry (FACS) for the determination of monocyte subset distribution was performed using a FACS Canto II with the FACS Diva Software (both Becton Dickinson) as shown in Figure 1. In brief, 100 μl of EDTA‐anticoagulated whole blood was stained with saturating concentrations of the following fluorochrome‐conjugated mAbs: phycoerythrin‐Cy7 (PE‐Cy7)‐labeled mAb for CD45 (eBioscience, Ref#25‐0459‐42, Clone HI30; 0.125μg/test), peridinin chlorphyll protein (PerCP)‐labeled mAb for CD14 (BD Biosciences, Ref#345786, Clone MφP9, 0.1μg/test), allophycocyanin (APC)‐H7‐labeled mAb for CD16 (BD Biosciences, Ref#560715; Clone 3G8; 5 μl/test,), phycoerythrin (PE)‐labeled mAb for CCR‐2 (R&D Systems, Ref#FAB151P, 5 μl/test) and corresponding isotype controls. After incubation for 15 min in the dark, 1.5 ml lysing solution (BD FACS Lysing solution, BD Biosciences, Ref#349202)) was added for additional 15 min. After 3 washing steps (in PBS, 820 RPM, 5 min), cells were resuspended in 1 ml PBS for FACS analysis. Monocytes were defined as CD45‐positive cells exhibiting a typical forward and sideward scatter profile and were distinguished into 3 subsets and defined as CM (CD14^++^CD16^‐^), IM (CD14^++^CD16^+^CCR2^+^) and NCM (CD14^+^CD16^++^CCR2^‐^; Fig. 1). Absolute counts were calculated by number of total leukocytes in relationship to CD45^+^ cells in flow cytometry. CM, IM, and NCM are given as percentage of total monocytes if not stated otherwise.

### 2.4 Statistical analysis

The primary endpoint of this study was survival at 6 months and the secondary endpoint was neurological outcome as determined by cerebral performance category (CPC)‐score at 6 months. Sample size calculation analysis revealed that in a cohort with a mortality rate of 50%, given a power of 0.8 and a significance level of 0.05, we would need 50 patients to detect an absolute difference of 3.5% between monocyte subsets of survivors and non‐survivors. To account for an anticipated dropout rate of 6%, we aimed to include 53 patients.

Categorical variables are summarized as counts and percentages and are compared by the χ^2^ or by Fisher's exact test as appropriate. Continuous variables are expressed as median and interquartile range (IQR). For correlations, Spearman's rank order correlation coefficient was used. Data was compared by Mann‐Whitney test. Cox proportional hazard regression analysis was performed to assess the effect of monocyte distribution on survival. Due to limited number of endpoints, we were able to include only a limited number of variables in the regression analysis. Therefore, we calculated a “CPR‐model” that included the CPR‐variables with the highest association with survival in univariate analysis, namely time to ROSC and witnessed cardiac arrest and a “Clinical model” that included age and APACHE II score. Kaplan–Meier analysis (log‐rank test) was applied to verify the time‐dependent discriminative power of monocyte subsets. Two‐sided *P*‐values < 0.05 indicated statistical significance. SPSS 21.0 (IBM Corporation, Armonk, NY, USA) was used for all analyses.

## 3 RESULTS

### 3.1 Baseline characteristics

Baseline characteristics are presented in Table 1. The study cohort consisted of 53 consecutive patients with either out‐of‐hospital CA (*n* = 31, 58.5%) or in‐hospital CA (*n* = 22, 41.5%) and sustained ROSC. Median age was 64.5 (49.8‐74.3) years and 40 (75.5%) of patients were male. A shockable rhythm was present in 28 (52.8%) of patients and median time to ROSC was 20 (10.0‐42.5) min.

### 3.2 Monocyte subset distribution, clinical characteristics, and inflammatory markers

Monocyte subset distribution was determined by flow cytometry (Fig. 1) and monocytes were defined as CD45^+^ cells exhibiting a typical forward and sideward scatter profile. Monocyte subsets were distinguished according to their CD14, CD16, and CCR‐2 expression into CM (CD14^++^CD16^‐^), IM (CD14^++^CD16^+^CCR2^+^), and NCM (CD14^+^CD16^++^CCR2^‐^). At day 0, median number of CM was 262 IQR 161–546 cells/μl (92.7 IQR 88.9‐95.1% of total monocytes), of IM was 9.5 IQR 3.3‐19.9 cells/μl (2.9 IQR 1.6‐4.6% of total monocytes), and of NCM was 9.0 IQR 4.3‐21.0 cells/μl (3.7 IQR 1.7‐7.4% of total monocytes). At day 3, median number of CM was 360 IQR 215–510 cells/μl (89.0 IQR 81.9‐92.2% of total monocytes), of IM was 20.9 IQR 7.7‐56.0 cells/μl (5.8 IQR 2.0‐9.5% of total monocytes), and of NCM was 14.8 IQR 0.1‐32.1 cells/μl (4.3 IQR 1.0‐8.4% of total monocytes). To test whether subset analysis without inclusion of CCR2 changes the main results, we also performed an analysis based solely on CD14 and CD16 expression, which shows comparable results (Supplementary Fig. S1).

Monocyte subset distributions at days 0 and 3 did not correlate with age, gender, or body mass index (BMI). In addition, monocyte subset distributions at days 0 and 3 were not associated with dosage of arterenol and the use of dobutamine at ICU‐admission.

CM (percentage of total monocytes) correlated inversely with both C‐reactive protein (CRP) and procalcitonin (PCT) at admission (CRP: *R* = ‐0.37, *P* = 0.015; PCT *R* = ‐0.37, *P* = 0.017) as well as with PCT after 72 h (*R* = ‐0.42, *P* = 0.014). IM showed a significant correlation with PCT at admission (*R* = 0.39, *P* = 0.011) whereas proportions of NCM did not correlate with inflammatory markers at admission nor after 72 h. Of interest, the disease severity score APACHE II at admission correlated with relative numbers of IM 72 h after admission (*R* = 0.436; *P* = 0.004).

### 3.3 Monocyte subset distribution and 6‐month mortality

**FIGURE 2:**
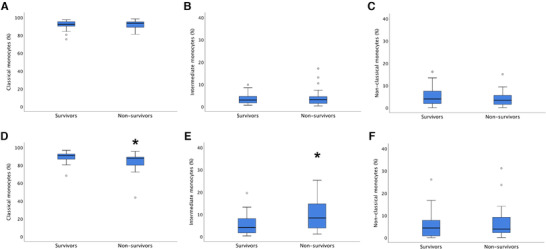
**Monocyte subset distribution and survival**. (**A–C**) Monocyte subset distribution according to mortality upon admission. Given are percentages of total monocytes for classical monocytes (**A**), intermediate monocytes (**B**), and non‐classical monocytes (**C**) as obtained by flow cytometry. No differences in monocyte subset distribution according to survival status 6 months after cardiac arrest were seen. (**D–F**) Monocyte subset distribution according to mortality 72 h after admission. Given are percentages of total monocytes for classical monocytes (**D**), intermediate monocytes (**E**), and non‐classical monocytes (**F**) as obtained by flow cytometry. Patients dying within 6 months after suffering from a cardiac arrest were characterized by a significantly lower percentage of classical monocytes (**D**) and a significantly higher percentage of intermediate monocytes (**E**) 72 h after admission. ^*^ *P* < 0.05

All‐cause mortality at 6 months post CA was 50.9% (27 out of 53 patients). Monocyte subset distribution at admission was not different in non‐survivors and survivors after 6 months (Fig. 2A–C). In survivors, monocyte subset distribution did not change between day 0 and day 3 in CM (‐0.7% IQR ‐4.7 to +2.3%; *P* = 0.45), nor in IM (‐2.7 IQR ‐51.3 to +130.9%; *P* = 0.86) or NCM (‐4.2% IQR ‐80.3 to +179.9%; *P* = 0.97). In contrast, non‐survivors showed a significant decrease of CM (‐7.7% IQR ‐12.8 to ‐3.3%; *P* = 0.015) and a significant increase of IM (+136.1% IQR +36.7 to +522.6%; *P* = 0.006) whereas NCM did not change significantly between day 0 and day 3 (+81.8% IQR ‐39.4 to +244.9%; *P* = 0.50). Subsequently, patients that died within 6 months showed a significantly lower proportion of CM (87.5% IQR 79.9–89.0% vs. 90.8% IQR 85.9–92.7%; *P* = 0.036) as compared to survivors (Fig. 2C–E). IM were significantly higher in non‐survivors (8.3% IQR 3.8–14.6% vs. 4.1% IQR 1.5–8.2%; *P* = 0.025), whereas proportion of NCM was not different in non‐survivors (3.8% IQR 2.2–9.1%) and survivors (4.3% IQR 0.7–7.8%; *P* = 0.67). Similar results were obtained when using absolute monocyte numbers, except that CM were not significantly different between non‐survivors and survivors at day 3 (data not shown).

**FIGURE 3:**
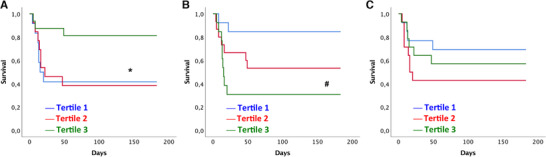
**Tertiles of monocyte subsets according to 6‐month survival**. Given are tertiles of monocyte subsets in percentages of total monocytes for classical monocytes (**A**), intermediate monocytes (**B**) and non‐classical monocytes (**C**) according to survival within 6 months after cardiac arrest. ^*^ *P* < 0.05 of tertile 1 and tertile 2 versus tertile 3. ^#^ *P* < 0.05 for tertile 3 versus tertile 1 and 2

Figure 3 shows Kaplan‐Meier survival curves of tertiles of monocyte subsets at day 3. A low proportion of circulating CM was associated with a high mortality. The hazard ratios (HR) for the first and second tertiles of CM were 4.3 (95% confidence interval (CI) 1.1‐16.6; *P* = 0.036) and 4.1 (95% CI 1.1‐15.3; *P* = 0.038) when compared to the third tertile. In contrast, a high proportion of IM was associated with increased mortality and the HR for the third tertile of IM showed a HR of 6.7 (95% CI 1.4–31.5; *P* = 0.015). The third tertile of IM was significantly associated with 6‐month all‐cause mortality after multivariate adjustment for time to ROSC and witnessed cardiac arrest with a HR of 6.4 (95% CI 1.6–37.9; *P* = 0.012; Table 2). In addition, the third tertile of IM was associated with outcome independent from age with a HR of 4.8 (95% CI 1.0–23.4; *P* = 0.05). However, intermediate monocyte distribution at day 3 was not significantly associated with survival after additional adjustment for disease severity at admission using the APACHE II score (Table 2).

**TABLE 2:** Multivariate analyses of association between intermediate monocytes after 72 hours and total mortality after 6 months

	Hazard ratio	95% CI	*P*‐value
**Unadjusted**			
First tertile	1		
Second tertile	3.7	0.8 – 18.0	0.101
Third tertile	6.7	1.4 – 31.5	0.015
**CPR model: Adjusted for time to ROSC and witnessed cardiac arrest**			
First tertile	1		
Second tertile	3.5	0.94–22.0	0.088
Third tertile	6.4	1.6–37.9	0.01
**Clinical model: Adjusted for age and APACHE II score at admission**			
First tertile	1		
Second tertile	2.3	0.42 – 12.2	0.33
Third tertile	3.2	0.62 – 17.1	0.17

CI, confidence interval; ROSC, return of spontaneous circulation; APACHE, acute physiology and chronic health evaluation.

### 3.4 Monocyte subset distribution and 6‐month neurological outcome

Overall 37.7% Patients (*n* = 20) had favorable neurological outcome (CPC 1–2), whereas 62.3% (*n* = 33) had a poor neurological outcome (CPC 3–5). Of interest, the relative change of IM between day 0 and day 3 (*R* = 0.39; *P* = 0.019) and the percentage of IM at day 3 (*R* = 0.32; *P* = 0.043) correlated with the CPC score at 6 months, however the relative change (*R* = ‐0.32; *P* = 0.068) and the percentage of CM at day 3 (*R* = ‐0.26; *P* = 0.11) and the percentage of NCM (*R* = 0.023; *P* = 0.89) did not correlate with neurological outcome. In contrast to monocyte subset distribution, the inflammatory markers CRP and PCT did neither correlate with six‐month mortality nor with neurological outcome at 6 months.

## 4 DISCUSSION

In the present prospective, observational study including 53 consecutive patients admitted to a medical ICU after ROSC, monocyte subset distribution was associated with 6‐month survival. Interestingly, monocyte subsets at ICU admission did not differ according to outcome. However, non‐survivors showed a significant decrease of CM and a significant increase of IM between admission and day 3. Subsequently, patients who did not survive the first 6 months were characterized by a skewed monocyte subset distribution with a significantly higher proportion of the pro‐inflammatory subset of IM and a lower proportion of CM 72 h after the CA. In contrast, the non‐classical subset did not differ between survivors and non‐survivors. When patients were stratified into tertiles of monocyte subsets, patients in the highest tertile of IM were at a 6.4‐fold higher risk of dying irrespective of time to ROSC and whether they had a witnessed CA.

We decided to include a mixed cohort of patients both after surviving an out‐of‐hospital CA as well as after in‐hospital CA. All patients experienced sustained ROSC before admission. Most patients were male, had a witnessed CA, and received bystander CPR. Patients suffering from OHCA and a shockable rhythm were more likely to survive, while those with higher lactate levels at admission and longer time to ROSC were more likely to die within 6 months, as expected from previous experience.^20^

The PCAS is a syndrome caused by whole body ischemia and reperfusion and involves ongoing myocardial dysfunction, a persisting cause of the initial arrest as well as severe brain injury and severe reperfusion syndrome.^3^ As myocardial failure and brain injury are thought to represent the main causes of death, therapeutic interventions and prognostication efforts mainly focus on those areas. However, inflammatory mechanisms have been implicated in myocardial reperfusion injury and thus might play an important role in whole body reperfusion injury.^21^, ^22^

As innate immune effector cells, monocytes may play an important role in the pathophysiology of PCAS.^23^ According to their surface expression pattern of CD14 and CD16, monocytes can be distinguished into at least three subtypes, namely CM (CD14^++^CD16^+^), IM (CD14^++^CD16^+^CCR2^+^), and NCM (CD14^+^CD16^++^CCR2).^8^ Initially, 2 subsets have been described, CD14^+^ CM and CD16^+^ NCM. The CD16^+^ subset accounts for ∼10% of circulating monocytes in healthy individuals and has been initially described as pro‐inflammatory. However, soon it became evident that there is considerable heterogeneity among CD16^+^ monocytes, and a third subset, the IM (CD14^++^CD16^+^) has been described.^8^, ^9^ Within the following years, it was shown that this intermediate subset, despite its name, is more than just a subset “in between.”^9^ This cell type is characterized by a strong inflammatory reaction upon stimulation and by an increased expression of surface molecules involved in reparative mechanisms.^24^, ^25^ Several clinical observations have described elevated IM as biomarkers for outcome and disease severity in critical conditions.^13^, ^15^, ^26^ In addition, large‐scale studies evaluated monocyte subsets as predictors of adverse cardiac outcomes in stable cohorts suggesting such a role for both the classical and intermediate subset.^11^, ^12^, ^27^

In our analysis, the proportion of CM at admission was inversely correlated with CRP levels, an unspecific marker of inflammatory activity as well as with PCT, a novel biomarker with potential to differentiate between inflammation and infection. In addition, IM showed a significant correlation with PCT levels at admission. It has to be noted that none of the included patients had clinical signs of sepsis. Previous studies however suggest a high proportion of bacteremia after CA, when specifically testing for it.^28^ We observed a strong correlation between the widely used disease severity score APACHE II at admission and the proportion of IM on day three after CA. This is in line with a small post cardiac surgery cohort.^29^ Therefore, one can speculate that severe organ dysfunction at admission leads to further activation of the innate immune system at day 3. When the APACHE II score was added to the multivariate regression analysis, the association between circulating IM and survival lost significance. Whether circulating monocytes therefore only represent disease severity upon admission or whether they further add specific detrimental effects cannot be answered from our data.

We were able to show that upon admission, monocyte subsets were not associated with 6‐month mortality. However, 72 h after admission, we could observe a remarkable shift toward an increase in IM accompanied by a decrease in CM. This fact is of major importance and scientific interest. One explanation for this observed “delay” in monocyte subset shift might be an impaired immune response in the first hours after CA as analyzed elsewhere.^30^ Another hypothesis may be that an initial shift toward an inflammatory monocyte subset distribution might be beneficial and a physiologic response of the organism towards the systemic ischemia and reperfusion. Those however, that fail to contain such an overactive monocyte response might develop a systemic inflammatory response syndrome leading to multi‐organ failure. These patients may be identified by elevated proportions of inflammatory monocytes in the days after the initial event. Several findings suggest a developmental relationship between monocyte subsets, with classicals released from the bone marrow, which subsequently differentiate into intermediates and finally NCM.^31^, ^32^ This might further explain our findings of prognostic abilities of monocyte subsets only after 72 h.

Several observations confirm our findings of a strong inflammatory activation in patients after CA, with 1 study analyzing monocyte behavior.^21^, ^22^, ^23^ The authors of the latter finding suggested changes in monocyte pattern recognition receptor signaling pathways and inflammasome activation may play a role in PCAS.^23^

Monocyte subsets were implicated in the pathophysiology of acute myocardial infarction and correlated with outcome and left ventricular function in those patients.^33^, ^34^, ^35^ The detailed mechanisms are not understood; therefore, one could speculate that the observed monocyte subset changes in our study might be due to myocardial injury. Conversely, the findings in AMI might be caused by reperfusion injury. Thus, our results might also be explained by a whole body reperfusion injury after ROSC in CA.

Another important aspect of PCAS and a common cause of death is severe brain damage. In our study, we could show a significant correlation between the proportion of IM and the CPC‐score at 6 months. This finding is of major importance, as the main goal for the patient and the health care system is discharge of patients with acceptable neurologic outcome. In this regard, monocyte subsets were described as predictors of defect size and outcome in other situations characterized by an acute and sudden brain injury, such as stroke.^36^, ^37^ In those studies, monocyte subsets were not only implicated as surrogate parameters of stroke size but more as reparative cells probably acting as a surrogate for a reparative process underway, which has been supported by experimental findings.^38^ Of interest, monocyte subsets have further been implicated in the deterioration of chronic degenerating neurologic diseases such as multiple sclerosis.^39^ Whether an increase of IM may reflect the size of neurologic damage or rather the reparative efforts underway cannot be answered by this analysis and warrants further mechanistic studies in experimental settings.

Further studies are strongly warranted to broaden our understanding of the pathophysiologic mechanisms involved in our findings and to design potential therapeutic interventions. Whether monocyte subset distribution constitutes a biomarker of an ongoing and escalating inflammatory activation or monocyte subsets have potential reparative effects or damaging effects is currently unknown. The recently published observation that IM exhibit a distinct micro‐RNA profile might be a first step in designing such an intervention.^25^

Several limitations of the current study need to be mentioned. First, this was a single‐center study including both patients after out‐of‐hospital and in‐hospital CA. Due to the rather small sample size, individual analysis of patient groups was not feasible. Still, for the treating ICU physician, it is of major interest whether a marker can predict outcome independent of case specifics. Further, larger studies are warranted to analyze whether monocyte subset distribution is involved in the pathophysiology of patients after both in‐ and out‐of‐hospital CA. As the present analysis is of observational nature, we can only describe an association between monocyte subset distribution and mortality but cannot draw any causality from our findings. In addition, only circulating monocyte subsets and routinely measured inflammatory markers were assessed, while other soluble mediators reflecting systemic inflammatory activation such as IL‐6 or IL‐1 were not measured in our population. Furthermore, blood was drawn within the first 24 h after ICU admission, which is why we cannot exclude time‐dependent variations within the first 24 h, which may render early monocyte subset measurements feasible for prognostication. Still, our findings suggest a later increase in IM being predictive of outcome.

In conclusion, we provide evidence for the predictive value of monocyte subset distribution for 6‐month survival in patients admitted to a medical ICU after surviving a CA independent from time to ROSC and witnessed cardiac arrest. Our results contribute further aspects on the role of the innate immune system in the pathophysiology of PCAS and may pose a therapeutic target.

## AUTHORSHIP

K.A.K. was associated with study conceptualization, methodology, validation, formal analysis, investigation, data curation, and wrote the original draft of the manuscript. M.L. was associated with investigation, methodology, data curation, conceptualization, and reviewed and edited the final manuscript. B.R. was associated with investigation, data curation, and reviewed and edited the final manuscript. P.J.H. was associated with formal analysis, investigation, data curation, and reviewed and edited the final manuscript. S.P.K. methodology, data curation, and reviewed and edited the final manuscript. A.M. was associated with methodology, data curation, reviewed and edited the final manuscript. K.H. was associated with funding acquisition, data curation, validation, and reviewed and edited the final manuscript. C.H. was associated with project administration, funding acquisition, and reviewed and edited the final manuscript. J.W. was associated with project administration, funding acquisition, validation, and reviewed and edited the final manuscript. G.H. was associated with study supervision, methodology, validation, conceptualization, and reviewed and edited the final manuscript. W.S.S. was associated with study supervision, project administration, funding acquisition, formal analysis, validation, and wrote the original draft of the manuscript.

## DISCLOSURE

The authors declare that they have no conflict of interest.

## Supporting information

Supporting Information
