# Supplementary material for: Monocyte subsets predict mortality after cardiac arrest
Source: J Leukoc Biol. 2020 Oct 5;109(6):1139–46. doi: 10.1002/JLB.5A0420-231RR (PMC8247267; doi:10.1002/JLB.5A0420-231RR)
Supplement: Supplementary file 1 — Supporting Information [file JLB-109-1139-s001.docx]

**Supplemental material for Krychtiuk et al “Monocyte subsets predict mortality after cardiac arrest”**

**Analysis of monocyte subsets and 6 month survival based solely on CD14 and CD16 expression**

To test whether subset analysis without inclusion of CCR2 changes the main results we also performed an analysis based solely on CD14 and CD16 expression, which shows comparable results. On admission, monocyte subset distribution was not associated with survival status at 6 months (CM (90.8 (IQR 85.9-94.4)% vs. 89.6 (IQR 77.6-93.4)%; n.s.); IM (5.6 (2.4-10.0)% vs. 5.4 (IQR 4.2-14.8)%, n.s.); NCM (3.7 (IQR 3.0-5.9)% vs. 4.2 (IQR 3.0-6.2)%; n.s., for survivors vs. non-survivors, respectively). Patients that died within six months showed a trend towards lower proportion of CM (84.7% IQR 72.4–91.0% vs. 87.9.8% IQR 82.7–91.3%; n.s.) and NCM (3.8 (1.8-6.8)% vs. 6.5 (3.3-8.6)%; n.s.) as compared to survivors on day 3. IM were significantly higher in non-survivors (10.9% IQR 7.0–22.8% vs. 5.7% IQR 3.6–8.9%; p=0.017) (Supplemental Figure 1).


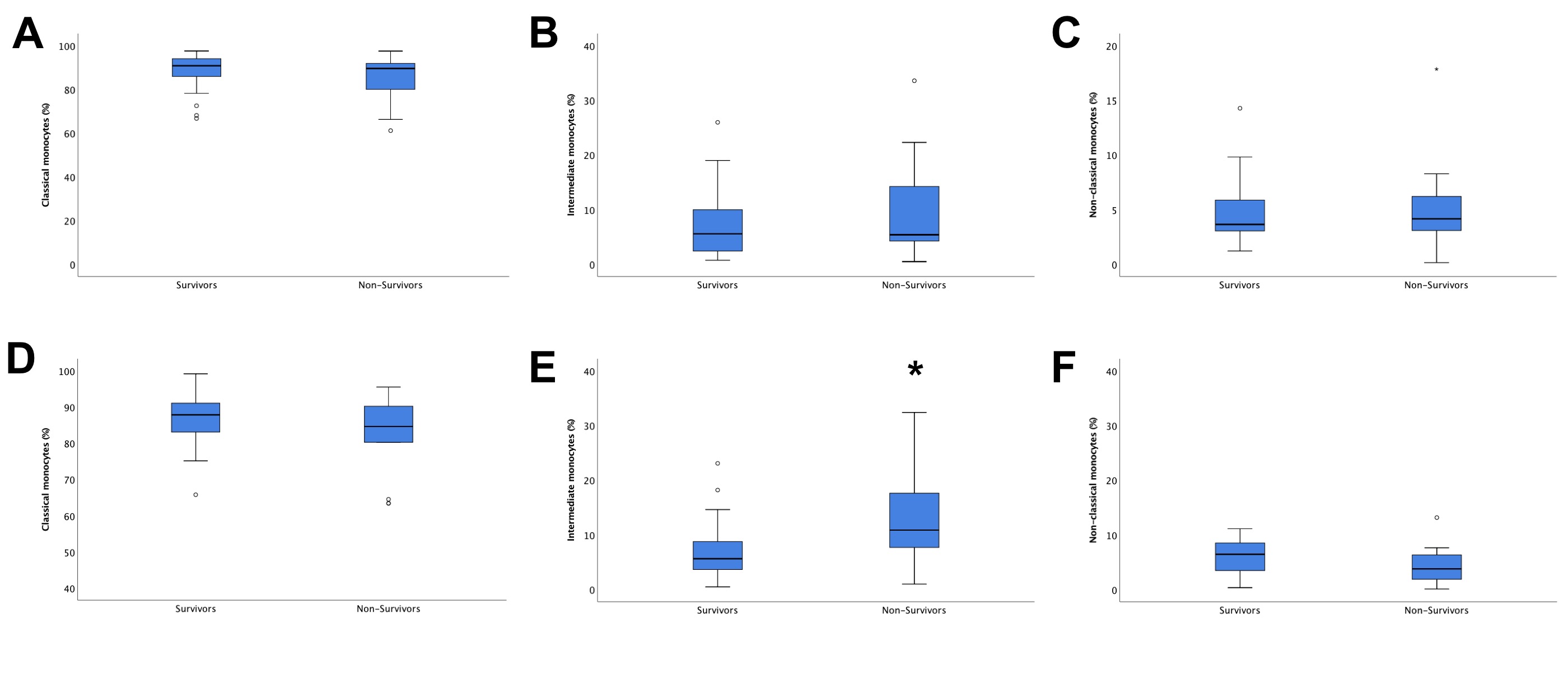


**Supplemental Figure 1 Monocyte subset distribution and survival, as analysed based solely on CD14 and CD16 expression without inclusion of CCR-2**

**A-C Monocyte subset distribution according to mortality upon admission.** Given are percentages of total monocytes for classical monocytes (A), intermediate monocytes (B) and non-classical monocytes (C) as obtained by flow cytometry. No differences in monocyte subset distribution according to survival status 6 months after cardiac arrest were seen.

**D-F Monocyte subset distribution according to mortality 72 hours after admission**

Given are percentages of total monocytes for classical monocytes (D), intermediate monocytes (E) and non-classical monocytes (F) as obtained by flow cytometry. Patients dying within 6 months after suffering from a cardiac arrest were characterized by a significantly higher percentage of intermediate monocytes (E) 72 hours after admission. * p<0.05
